# Comparative Analysis of Serum microRNA in Diagnosed Ocular Sarcoidosis versus Idiopathic Uveitis with Ocular Manifestations of Sarcoidosis

**DOI:** 10.3390/ijms231810749

**Published:** 2022-09-15

**Authors:** Shoko Saito, Hiroshi Keino, Ichiro Takasaki, Shinya Abe, Hideo Kohno, Kousuke Ichihara, Isami Hayashi, Makiko Nakayama, Yukihiro Tsuboshita, Sawako Miyoshi, Susumu Okamoto, Annabelle A. Okada

**Affiliations:** 1 Department of Ophthalmology, Kyorin University School of Medicine, 6-20-2 Shinkawa, Tokyo 181-8611, Japan; 2 Department of Pharmacology, Graduate School of Science and Engineering for Research, University of Toyama, 3190 Gofuku, Toyama 930-8555, Japan; 3 Department of Ophthalmology, Graduate School of Medicine and Pharmaceutical Sciences, University of Toyama, 2630 Sugitani, Toyama 930-0194, Japan; 4 Department of Ophthalmology, Jikei University School of Medicine, 3-25-8 Nishishinbashi, Tokyo 105-8461, Japan; 5 Department of Medical Technology, Faculty of Health Sciences, Kyorin University, 5-4-1 Shimorenjyaku, Tokyo 181-8611, Japan; 6 Department of General Medicine, Kyorin University School of Medicine, 6-20-2 Shinkawa, Tokyo 181-8611, Japan

**Keywords:** idiopathic uveitis, sarcoidosis, microRNA, serum, bioinformatics

## Abstract

“Idiopathic” is the most common category of uveitis, representing cases in which a specific diagnosis has not been established despite work-up. Sarcoidosis is a systemic granulomatous disorder affecting multiple organs including the lungs, skin, kidneys, and eyes. We used microRNA (miRNA) microarrays to investigate serum miRNA profiles of patients with ocular sarcoidosis as diagnosed by specific criteria (diagnosed ocular sarcoidosis), and patients with idiopathic uveitis characterized by ocular manifestations of sarcoidosis (suspected ocular sarcoidosis). Principal component analysis (PCA) and hierarchical clustering showed that serum miRNA profiles of diagnosed ocular sarcoidosis and suspected ocular sarcoidosis were both clearly distinguishable from healthy controls. Furthermore, comparative analysis of the miRNA profiles showed highly similar patterns between diagnosed ocular sarcoidosis and suspected ocular sarcoidosis. Pathway analysis revealed common pathways were involved in the two groups, including those of WNT signaling and TGF-beta signaling. Our study demonstrated a high overlap of differentially expressed serum miRNAs in patients with diagnosed ocular sarcoidosis and suspected ocular sarcoidosis, suggesting that these groups share a similar underlying pathology and may represent possible variants of the disease. Characterization of serum miRNA profiles may provide an opportunity for earlier diagnosis and treatment, and may inform more accurate clinical prognosis in patients with an ocular sarcoidosis phenotype.

## 1. Introduction

Uveitis can cause significant visual loss [1], and its etiology includes both infectious and non-infectious causes. The application of new diagnostic tools including multimodal ocular imaging, laboratory testing, and multiplex polymerase chain reaction (PCR) testing of intraocular fluids has contributed greatly to the diagnosis of specific uveitic entities. However, “idiopathic”, representing cases in which a specific diagnosis has not been established despite work-up, remains the most common category of uveitis. In national surveys in Japan, idiopathic uveitis was found to account for 35–40% of all patients with uveitis and this proportion was unchanged over two decades [2,3,4]. In general, patients are categorized as having idiopathic uveitis after excluding known specific uveitic diseases by careful history taking, comprehensive ophthalmological examination, and ancillary laboratory testing as deemed appropriate.

Sarcoidosis is a systemic granulomatous disorder affecting multiple organs including the lungs, skin, kidneys, and eyes [5,6]. Ocular sarcoidosis is the most common specific diagnosis for patients with uveitis in Japan [4], and currently the diagnosis is widely based on criteria proposed by the International Workshop on Ocular Sarcoidosis (IWOS) [7,8]. However, uveitis specialists have also experienced patients with granulomatous ocular findings identical to those with ocular sarcoidosis diagnosed by the IWOS criteria, but in whom ancillary testing did not meet the IWOS criteria for diagnosis. Indeed, it has been reported that 54% of patients with unclassified (idiopathic) uveitis had the IWOS ocular clinical signs for “probable” ocular sarcoidosis based on the presence of three or more of seven ocular clinical signs. A recent study demonstrated that gene expression profiles in peripheral blood may be helpful in determining specific diagnoses in patients with idiopathic uveitis, and this may have important therapeutic and prognostic implications [9].

There are circulating cell-free nucleic acids released into the serum/plasma by various tissues and cells, including DNA, RNA, microRNA (miRNA), and long-noncoding RNA (lncRNA) [10,11]. Recent studies have suggested that circulating cell-free nucleic acids may play a role as epigenetic biomarkers for the diagnosis, classification, management, and treatment of various diseases including malignancies and autoimmune disorders [10,12]. MiRNAs are small noncoding RNAs that regulate gene expression at the post-transcriptional level [13]. Several investigators have reported on the feasibility of using miRNAs as biomarkers for the diagnosis of non-infectious uveitis, and miRNAs may also advance our understanding of the pathogenic mechanisms involved in these diseases [14,15,16,17].

We conducted a multicenter study to investigate serum miRNA profiles in patients with diagnosed ocular sarcoidosis and patients with idiopathic uveitis characterized by the presence of ocular manifestations of sarcoidosis, as well as in healthy controls. We were able to identify a shared miRNA profile in the two patient groups, and also examined the feasibility of machine learning using leave-one-out cross-validation (LOOCV) to classify patients based on miRNA expression profile.

## 2. Results

### 2.1. Demographics

The demographic characteristics of uveitis patients (*n* = 21) and healthy control subjects (*n* = 16) who participated in this study are summarized in Table 1. Eleven of the patients were diagnosed with ocular sarcoidosis by IWOS criteria (hereafter referred to as “diagnosed ocular sarcoidosis”) and 10 were patients in whom at least three of seven IWOS ocular clinical signs were present but did not meet the criteria for diagnosis (hereafter referred to as “suspected ocular sarcoidosis”). There was no significant difference in age distribution between the three groups (two uveitis groups and healthy controls). All 21 patients had bilateral uveitis and all had panuveitis. Of the 21 patients, 15 were women and 6 were men. There was no significant difference regarding sex distribution between the three groups. The mean soluble IL-2 receptor (sIL-2R) level and angiotensin-converting enzyme (ACE) level were significantly higher in diagnosed ocular sarcoidosis patients compared to patients with suspected ocular sarcoidosis. Nine of the eleven patients (82%) with diagnosed ocular sarcoidosis had evidence of bilateral hilar lymphadenopathy on radiographic examination of the chest, whereas none of the patients with suspected ocular sarcoidosis had this finding.

### 2.2. Differential Expression of Serum miRNAs and Pathway Analysis in Patients with Diagnosed Ocular Sarcoidosis versus Healthy Controls

**Figure 1:**
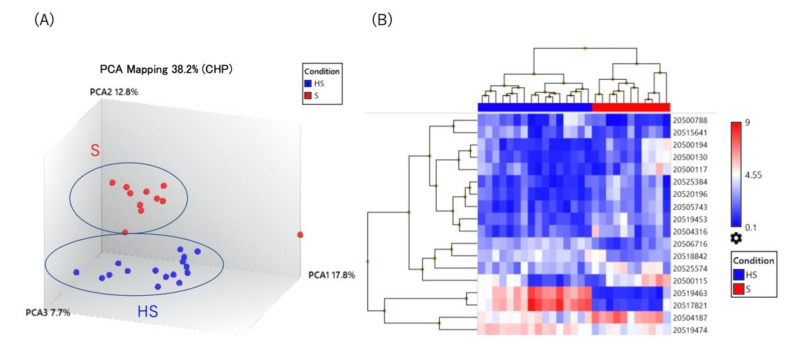

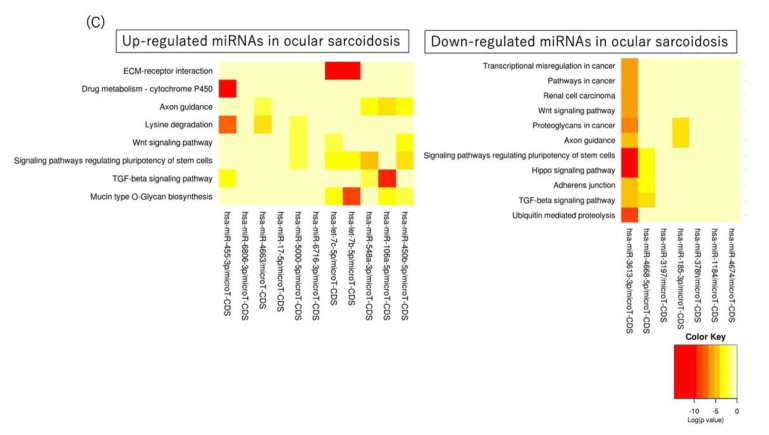
(**A**) Principal component analysis, (**B**) unsupervised hierarchical clustering analysis, (**C**) pathway analysis using serum miRNAs in diagnosed ocular sarcoidosis versus healthy controls. HS: healthy subjects, S: patients with diagnosed ocular sarcoidosis.

We employed GeneChip^®^ miRNA 4.0 arrays to compare the expression levels of 2578 mature miRNAs in the serum of patients with diagnosed ocular sarcoidosis and healthy controls. As shown in Figure 1A,B, both principal component analysis (PCA) and unsupervised hierarchical clustering displayed distinctly different profiles between the two groups. A total of 18 miRNAs were significantly different (*p* < 0.05), either up-regulated (11 miRNAs with >2.0-fold change) or down-regulated (7 miRNAs with <0.5-fold change), in diagnosed ocular sarcoidosis compared to healthy controls (Table 2). In order to identify possible target pathways of the differentially expressed miRNAs, we performed Kyoto Encyclopedia of Genes and Genomes (KEGG) pathway enrichment analysis with the online tool DNA intelligent analysis (DIANA)-mirPath. The results displayed in Figure 1C show that the pathways targeted by up- or down-regulated miRNAs in patients with diagnosed ocular sarcoidosis involve the wingless/integrated (WNT) signaling pathway, signaling pathways regulating the pluripotency of stem cells, and the transforming growth factor (TGF)-beta signaling pathway.

### 2.3. Differential Expression of Serum miRNAs and Pathway Analysis in Patients with Suspected Ocular Sarcoidosis versus Healthy Controls

**Figure 2:**
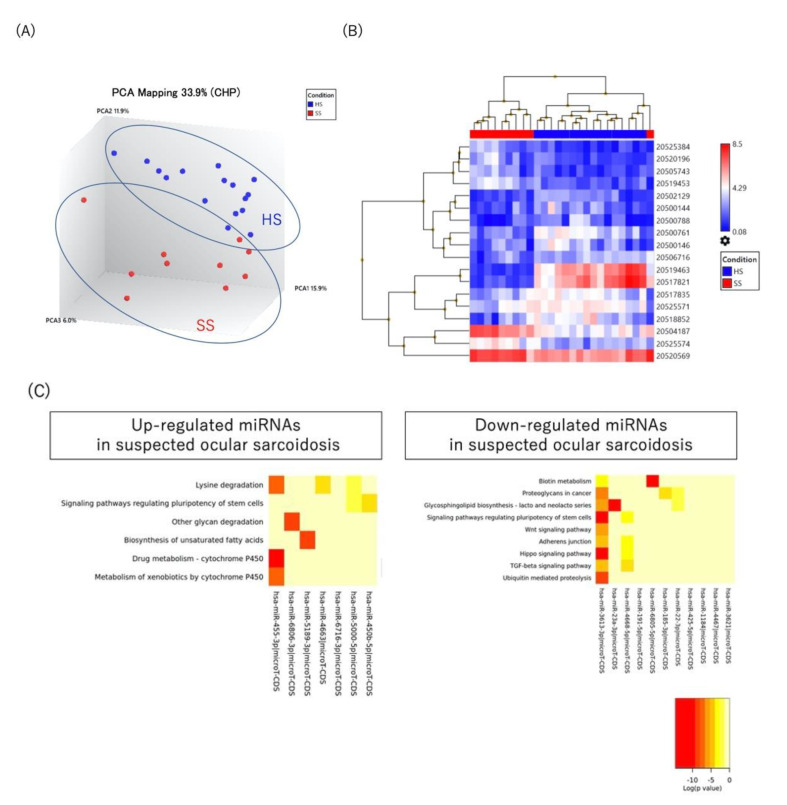
(**A**) Principal component analysis, (**B**) unsupervised hierarchical clustering analysis, (**C**) pathway analysis using serum miRNAs in suspected ocular sarcoidosis versus healthy controls. HS: healthy subjects, SS: patients with suspected ocular sarcoidosis.

As shown in Figure 2A,B, both PCA and unsupervised hierarchical clustering revealed distinctly different profiles in patients with suspected ocular sarcoidosis versus healthy controls. A total of 18 miRNAs differed significantly (*p* < 0.05), either up-regulated (7 miRNAs with >2.0-fold change) or down-regulated (11 miRNAs with <0.5-fold change), in suspected ocular sarcoidosis compared to healthy controls (Table 3). Figure 2C shows the pathways targeted by the up- or down-regulated miRNAs in suspected ocular sarcoidosis, and they include signaling pathways regulating the pluripotency of stem cells and the TGF-beta signaling pathway. In addition, drug metabolism by cytochrome P450 and the metabolism of xenobiotics by P450 were detected as pathways targeted by up-regulated miRNAs in suspected ocular sarcoidosis.

### 2.4. Differential Expression of Serum miRNAs in Diagnosed Ocular Sarcoidosis versus Suspected Ocular Sarcoidosis

**Figure 3:**
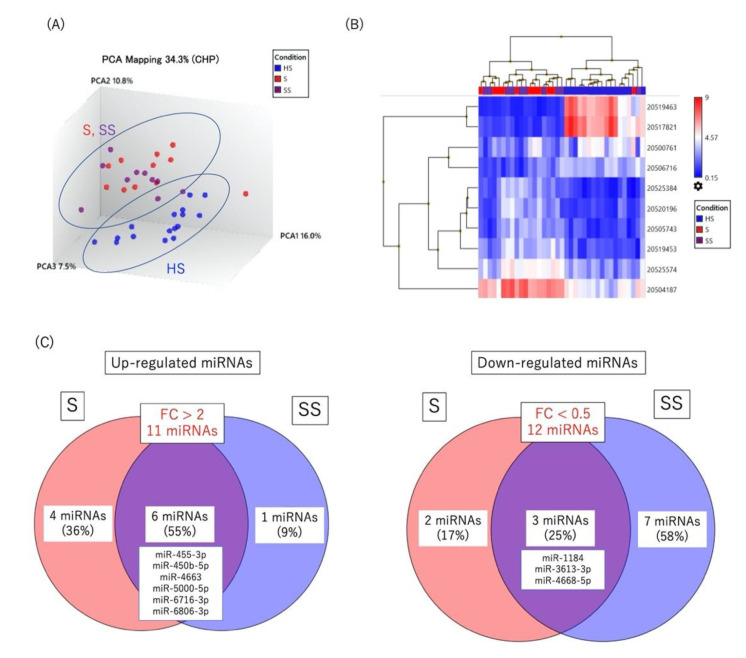
(**A**) Principal component analysis, (**B**) unsupervised hierarchical clustering analysis, (**C**) Venn diagrams showing miRNAs significantly up-regulated (>2.0-fold change) or down-regulated (<0.5-fold change) in diagnosed ocular sarcoidosis or suspected ocular sarcoidosis compared to healthy control subjects (*p* < 0.05). FC: fold change, HS: healthy subjects, S: diagnosed ocular sarcoidosis, SS: suspected ocular sarcoidosis.

As shown in Figure 3A,B, although both PCA and unsupervised hierarchical clustering revealed distinctly different profiles in patients with either diagnosed or suspected ocular sarcoidosis compared to healthy controls, there was marked overlap between patients with diagnosed ocular sarcoidosis and patients with suspected ocular sarcoidosis. The Venn diagrams in Figure 3C display a comparison of significantly up- or down-regulated miRNAs in these two patient groups. Six miRNAs (55%) were found to be up-regulated in both patient groups, four miRNAs were exclusively up-regulated in patients with diagnosed ocular sarcoidosis, and one miRNA was exclusively up-regulated in patients with suspected ocular sarcoidosis. On the other hand, three miRNAs (25%) were found to be down-regulated in both patient groups, two miRNAs were exclusively down-regulated in patients with diagnosed ocular sarcoidosis, and seven miRNAs were exclusively down-regulated in patients with suspected ocular sarcoidosis.

### 2.5. Classification of Patients by Machine Learning

Using our data on serum miRNA expression profiles, we investigated the prediction performance of machine learning to classify the study patients using random forest algorithms with the classification performance tested by the “leave-one-out” cross-validation (LOOCV) method. As shown in Table 4, the random forest algorithms had moderate accuracy in discriminating between diagnosed versus suspected ocular sarcoidosis, with only three patients with diagnosed ocular sarcoidosis and three patients with suspected ocular sarcoidosis being misclassified. The sensitivities and specificities were 64% and 88%, respectively, for predicting diagnosed ocular sarcoidosis, and 64% and 89%, respectively, for predicting suspected ocular sarcoidosis.

## 3. Discussion

As uveitis specialists, we sometimes encounter pediatric patients with chronic iridocyclitis that appears identical to the type of uveitis observed in patients diagnosed with juvenile idiopathic arthritis, but without any arthritis signs or symptoms [9,18]. Similarly, we often see patients with intraocular inflammation that appears typical for patients diagnosed with ocular sarcoidosis, but in whom the results of laboratory testing do not fulfill the IWOS diagnostic criteria [8]. In both of these situations, gene expression profiling of peripheral blood has the potential to inform us of the pathogenic mechanisms of disease, and may allow earlier diagnosis for some patients [19]. Moreover, the results of gene profiling may provide valuable insights into deciding therapy based on the specific mechanism of disease. In the present study, we analyzed serum miRNA profiles of patients diagnosed with ocular sarcoidosis using the IWOS criteria (diagnosed ocular sarcoidosis), patients who did not meet the IWOS criteria but had a similar pattern of intraocular inflammation (suspected ocular sarcoidosis), and healthy controls. The miRNA profile was distinctly different between diagnosed ocular sarcoidosis and healthy controls, as well as between suspected ocular sarcoidosis and healthy controls. Furthermore, PCA and hierarchical clustering showed that the miRNA patterns were highly similar in diagnosed ocular sarcoidosis and suspected ocular sarcoidosis. KEGG pathway analysis revealed common mechanistic pathways that may be involved in the pathogenesis of both diagnosed and suspected ocular sarcoidosis. Compared to healthy controls, 55% of the same miRNAs were up-regulated and 25% of the same miRNAs were down-regulated in both groups.

Rosenbaum and colleagues developed a machine learning algorithm to aid in reclassifying idiopathic uveitis based on peripheral whole blood gene expression profiles. Using the algorithm, the diagnostic likelihood of four specific uveitic diseases associated with systemic manifestations (one was sarcoidosis) was assessed in 38 patients with idiopathic uveitis, and the results suggested reclassification of 11 of the patients [9]. Although our study did not generate likelihood statistics, 3 of 10 patients with suspected ocular sarcoidosis were classified as having ocular sarcoidosis by our machine learning algorithm, suggesting a similarity in miRNA signature between patients with suspected ocular sarcoidosis and patients with IWOS criteria-diagnosed ocular sarcoidosis. Furthermore, such miRNA profiles may serve as biomarkers in understanding the biological processes involved in sarcoidosis and aid in assessing treatment outcomes. Finally, the use of miRNA profiling may open up an opportunity to establish diagnoses in our pool of “idiopathic uveitis” patients.

The present study compared the serum miRNA signatures of patients with either diagnosed or suspected ocular sarcoidosis versus healthy controls. PCA and unsupervised hierarchical clustering displayed a distinct profile between diagnosed ocular sarcoidosis and healthy controls. By KEGG pathway analysis of associated miRNAs, 8 pathways appeared to be potentially up-regulated and 11 pathways appeared to be potentially down-regulated in diagnosed ocular sarcoidosis. Our results are consistent with a recent report [16], demonstrating that serum miRNA profiles of ocular sarcoidosis were clearly separate from those of healthy controls, and pathway analysis of down-regulated serum miRNAs included 10 of the 11 pathways we identified in our study. Although the source of circulating miRNAs in the serum of patients with ocular sarcoidosis remains unknown, peripheral blood cells, lung tissue, and intraocular granulomatous tissue may be involved in the secretion of these circulating miRNAs. In a study of patients with pulmonary sarcoidosis, Crouser and colleagues found that miRNA profiles in lung tissue and peripheral blood mononuclear cells targeted the TGF-beta/WNT signaling pathways [20]. We also found that the WNT signaling and TGF-beta signaling pathways were related to up- and/or down-regulated miRNAs in both patients with diagnosed ocular sarcoidosis and patients with suspected ocular sarcoidosis. Previous studies have demonstrated that the TGF-beta/WNT pathways may be involved in the pathogenesis of sarcoidosis [20,21,22]. Taken together, these findings suggest that, not only peripheral blood cells/lung tissue, but also intraocular granulomatous tissue may be contributing to the secretion of circulating serum miRNAs in patients with ocular manifestations of sarcoidosis. Characterization of the serum miRNA signature may lead to further understanding of the underlying pathogenesis of the phenotype of ocular sarcoidosis.

This study has several limitations. First, the sample size of each group was relatively small. Second, confirmation by polymerase chain reaction of the differentially expressed miRNAs was not performed. Third, although all patients underwent chest X-ray evaluation to assess for possible pulmonary lesions consistent with sarcoidosis, only some patients had chest computed tomography performed. Therefore, some of our patients with suspected ocular sarcoidosis may in fact have fulfilled the IWOS criteria for full diagnosis if more radiographic testing had been pursued [23,24], Fourth, characterization of the serum miRNA profiles of patients with other phenotypes of idiopathic uveitis, such as non-granulomatous idiopathic uveitis, is necessary to further characterize the specific miRNA profiles of suspected ocular sarcoidosis. Fifth, the effect of age, gender, the duration of uveitis, laterality, and anatomical location of uveitis on serum miRNA profiles need to be considered. Finally, we did not investigate miRNA signature alterations over follow-up in patients with diagnosed ocular sarcoidosis and suspected ocular sarcoidosis, and further study is required to examine the impact of systemic treatment. Regardless, we believe the results of the present investigation show promise for this research approach in evaluating patients with idiopathic uveitis in an effort to establish a diagnosis that may potentially impact treatment decisions and ultimately visual outcomes. We plan further investigations in larger numbers of patients with diagnosed ocular sarcoidosis and suspected ocular sarcoidosis in order to validate this approach for clinical use in the future.

## 4. Materials and Methods

### 4.1. Ethics Statement

This study was conducted in accordance with the tenets of the Declaration of Helsinki, and was approved by the Institutional Review Boards of Kyorin University Hospital, Toyama University Hospital, and Jikei University Hospital (protocol code: 763 and date of approval: 30 January 2020). All patients and healthy control subjects provided written informed consent at the time of enrollment in the study.

### 4.2. Subjects

Peripheral blood samples (4 mL) were obtained between January 2020 and October 2021 from 11 patients with diagnosed ocular sarcoidosis, 10 patients with suspected ocular sarcoidosis, and 16 healthy control subjects. All patients had active intraocular inflammation (panuveitis or posterior uveitis) and were not receiving systemic immunosuppressive treatment at the time of serum collection. The healthy control subjects were confirmed by careful questioning to have no history of uveitis, systemic inflammatory disorders, malignancy, or diabetes mellitus. Serum was isolated from blood samples and stored at −80 °C prior to processing.

“Diagnosed ocular sarcoidosis” was defined as uveitis with features meeting the IWOS criteria for ocular sarcoidosis, and “suspected ocular sarcoidosis” was defined as idiopathic uveitis with at least 3 of 7 IWOS ocular clinical signs suggestive of ocular sarcoidosis but not meeting the criteria for ocular sarcoidosis [8]. The IWOS intraocular findings suggesting ocular sarcoidosis are as follows: (1) mutton-fat keratic precipitates and/or iris nodules, (2) tent-shaped peripheral anterior synechia and/or trabecular meshwork nodules, (3) vitreous opacities (snowballs/string-of-pearls), (4) multiple chorioretinal peripheral lesions (active or atrophic), (5) segmental or nodular periphlebitis and/or macroaneurysm, (6) optic disc nodule(s)/granuloma(s) and/or solitary choroidal nodule, and (7) bilaterality [7,25].

For the present study, the following data were collected from medical records: age at uveitis onset, sex, best-corrected visual acuity, laterality, anatomic type of uveitis, presence of IWOS intraocular findings, ocular complications, and ancillary test results. Anatomic type of uveitis was based on the Standardization of Uveitis Nomenclature (SUN) guidelines [1]. In general, diagnosis of specific uveitic diseases or systemic disease associations was based on detailed clinical history, extensive review of systems, complete ophthalmologic examination, and uveitis-directed laboratory testing when deemed necessary [26]. Blood tests included complete blood count, erythrocyte sedimentation rate, C-reactive protein, electrolytes, liver enzymes, creatinine, serum soluble interleukin-2 receptor (sIL-2R) level, angiotensin-converting enzyme (ACE) level, and syphilis serological test. Lysozyme in serum was not measured as this examination is not covered by the healthcare insurance system in Japan. Urine analysis, chest X-ray examination, tuberculin skin test, and electrocardiogram were routinely performed; however, chest computed tomography and interferon gamma release assay were only performed in selected patients. Patients were referred to pulmonology depending on the results of initial ancillary testing, and to dermatology for skin signs or symptoms.

### 4.3. Extraction of RNA from Serum Sample and Microarray Analysis

Since a recent study by Rosenbaum et al. demonstrated that transcriptional signatures from peripheral blood have the potential to aid in identifying specific subsets among patients with idiopathic uveitis, we hypothesized that a similar approach of “molecular characterization” using miRNAs could allow further classification of our patients with idiopathic uveitis [9]. Total RNA was extracted from serum samples using the Qiagen miRNeasy^®^ Mini Kit (#217184, Qiagen, GmbH, Hilden, Germany) following the manufacturer’s protocol. For the microRNA assay, a 200 μL aliquot of each serum sample was used. Total RNA was eluted into 14 μL of RNAse-free water and the concentration of RNA was analyzed by measuring a 2 µL aliquot on a NanoDrop ND-3300 Fluorospectrometer (Thermo Fisher Scientific, Waltham, MA, USA). The miRNA was then prepared using the FlashTag™ Biotin-HSA RNA Labeling Kit for microarray analysis (Thermo Fisher Scientific, Waltham, MA, USA). Biotin-labeled RNA targets were hybridized to GeneChip^®^ miRNA 4.0 arrays (Thermo Fisher Scientific, Waltham, MA, USA). Probe signal intensities were log_2_-transformed and normalized based on the total intensity of the arrays using Transcriptome Analysis Console (TAC) v4.0 (Thermo Fisher Scientific, Waltham, MA, USA) and quality check of each sample was performed using QC metrics view on TAC v4.0. PCA was performed using probe set intensity of each sample.

### 4.4. Cluster Analysis and Venn Diagrams

The miRNA probes with a >2.0-fold change or with a *p*-value < 0.05 between patients and healthy controls were used for filtering. Hierarchical clustering and Venn diagrams were generated for normalized samples.

### 4.5. Pathway Analysis

Differentially expressed miRNAs identified in the present study were mapped by DNA intelligent analysis (DIANA)-mirPath tool (https://dianalab.e-ce.uth.gr/html/mirpathv3/index.php?r=mirpath, accessed on 22 July 2022) to identify target pathways using Kyoto Encyclopedia of Genes and Genomes (KEGG) pathway analysis and a *p*-value < 0.01 was considered to be statistically significant for this analysis.

### 4.6. Machine Learning

Python 3.9.12 (DE, USA) was used to produce a machine learning model based on random forest classification with Boruta algorithm (0.30) in order to compare patients with diagnosed ocular sarcoidosis, patients with suspected ocular sarcoidosis and healthy controls. The random forest algorithm used the hyperparameters of number of trees = 5000, criterion = “gini”, max depth = none, mini sample lear = 1, and max features = “auto”. The Boruta algorithm used the hyperparameters of n estimators = “auto”, max iter = 100, and alpha = 0.05. The sensitivity and specificity of resulting classification scores were calculated. Leave-one-out cross-validation was employed to validate the model’s performance.

### 4.7. Statistical Analysis

The Shapiro–Wilk test was performed to evaluate data distribution. Continuous variables were compared between two groups using the Mann–Whitney U test or Student’s *t*-test and the ANOVA was used for comparison of 3 or more groups. Categorical variables were analyzed with the Fisher exact probability test or chi-square test. Statistical analyses were performed using SPSS version 28. A *p*-value of < 0.05 was considered to be statistically significant unless otherwise indicated.

## 5. Conclusions

Our study demonstrated a high overlap of the differential expression of serum miRNAs in patients with diagnosed ocular sarcoidosis and patients with suspected ocular sarcoidosis, suggesting that both groups share similar underlying pathogenic mechanisms. Further characterization of serum miRNA profiles may provide new insights into diagnosis, therapeutic decisions, and visual prognosis in patients with idiopathic uveitis that have the features of ocular sarcoidosis but do not meet current diagnostic criteria.

## Figures and Tables

**Table 1:** Demographics.

	Healthy Controls	Diagnosed Ocular Sarcoidosis	Suspected Ocular Sarcoidosis	*p*-Value
Number	16	11	10	
Age (mean ± SD)	57.1 ± 10.3	61.5 ± 13.0	59.4 ± 13.0	0.629 **
Age range (years)	36–71	36–75	36–74	
Bilateral uveitis	NA	11	10	ND
Panuveitis	NA	11	10	ND
Gender (men/women)	7/9	5/6	1/9	0.149 ¶
sIL-2R (U/mL) (141–394) *	NA	784.7 ± 278.8	387.2 ± 141.7	<0.001 ‡
ACE (U/L) (7–25) *	NA	28.5 ± 14.9	18.8 ± 4.7	0.043 ‡
BHL (present/absent)	NA	9/2	0/10	<0.001 ‡‡

ACE: angiotensin-converting enzyme, BHL: bilateral hilar lymphadenopathy, NA: not applicable, ND: not done, sIL-2R: soluble interleukin-2 receptor. SD: standard deviation. *: normal range. **: ANOVA, ¶: chi-square test, ‡: Student’s *t*-test, ‡‡: Fisher exact probability test.

**Table 2:** Differential expression of serum miRNAs in diagnosed ocular sarcoidosis versus healthy controls.

miRNA	Fold Change	*p*-Value
hsa-miR-455-3p	3.67	9.96 × 10^−6^
hsa-let-7b-5p	3.65	0.0072
hsa-miR-5000-5p	2.71	0.0003
hsa-miR-6716-3p	2.62	0.0003
hsa-miR-6806-3p	2.57	0.0012
hsa-miR-106a-5p	2.52	0.0015
hsa-miR-4663	2.51	3.74 × 10^−5^
hsa-miR-548a-3p	2.37	0.0015
hsa-miR-450b-5p	2.36	0.0002
hsa-miR-17-5p	2.33	0.0084
hsa-let-7c-5p	2.25	0.0458
hsa-miR-4674	−2.01	3.01 × 10^−5^
hsa-miR-185-3p	−2.05	0.0112
hsa-miR-378h	−2.12	0.0077
hsa-miR-3197	−2.19	0.0386
hsa-miR-1184	−2.82	1.95 × 10^−6^
hsa-miR-4668-5p	−41.1	3.42 × 10^−16^
hsa-miR-3613-3p	−41.96	5.33 × 10^−13^

**Table 3:** Differential expression of serum miRNAs in suspected ocular sarcoidosis versus healthy controls.

miRNA	Fold Change	*p*-Value
hsa-miR-455-3p	4.61	2.33 × 10^−10^
hsa-miR-4663	3.1	0.0001
hsa-miR-6806-3p	2.74	4.05 × 10^−5^
hsa-miR-450b-5p	2.71	0.0003
hsa-miR-5189-3p	2.42	6.62 × 10^−5^
hsa-miR-6716-3p	2.27	0.0002
hsa-miR-5000-5p	2.26	0.0024
hsa-miR-6805-5p	−2.03	0.0011
hsa-miR-22-3p	−2.03	0.0123
hsa-miR-23a-3p	−2.07	0.0435
hsa-miR-1184	−2.28	0.0021
hsa-miR-185-3p	−2.33	0.0248
hsa-miR-425-5p	−2.57	0.0006
hsa-miR-3621	−2.76	0.0006
hsa-miR-4467	−2.91	0.0004
hsa-miR-191-5p	−4.06	8.91 × 10^−6^
hsa-miR-3613-3p	−31.43	3.88 × 10^−11^
hsa-miR-4668-5p	−34.86	7.07 × 10^−15^

**Table 4:** Classification of uveitis patients by machine learning based on the serum miRNA profile through LOOCV.

	Predicted HS	Predicted S	Predicted SS
actual HS	16	0	0
actual S	1	7	3
actual SS	1	3	6

HS: healthy subjects, S: diagnosed ocular sarcoidosis, SS: suspected ocular sarcoidosis.

## Data Availability

Not applicable.
